# Development of Breeder-Friendly KASP Markers for Low Concentration of Kunitz Trypsin Inhibitor in Soybean Seeds

**DOI:** 10.3390/ijms22052675

**Published:** 2021-03-06

**Authors:** M. Luciana Rosso, Chao Shang, Qijian Song, Diana Escamilla, Jay Gillenwater, Bo Zhang

**Affiliations:** 1 School of Plant and Environmental Sciences, Virginia Tech, Blacksburg, VA 24061, USA; luciana@vt.edu (M.L.R.); chshang@vt.edu (C.S.); 2 Beltsville Agricultural Research Center, Soybean Genomics and Improvement Laboratory, USDA-ARS, Beltsville, MD 20705, USA; qijian.song@ars.usda.gov; 3 Department of Agronomy, Purdue University, West Lafayette, IN 47907, USA; descamil@purdue.edu; 4 Department of Crop and Soil Sciences, North Carolina State University, Raleigh, NC 27695, USA; jhgille2@ncsu.edu

**Keywords:** Kunitz trypsin inhibitor, soybean, quantitative trait loci, anti-nutritional factor, single nucleotide polymorphism, KASP

## Abstract

Trypsin inhibitors (TI), a common anti-nutritional factor in soybean, prevent animals’ protein digestibility reducing animal growth performance. No commercial soybean cultivars with low or null concentration of TI are available. The availability of a high throughput genotyping assay will be beneficial to incorporate the low TI trait into elite breeding lines. The aim of this study is to develop and validate a breeder friendly Kompetitive Allele Specific PCR (KASP) assay linked to low Kunitz trypsin inhibitor (KTI) in soybean seeds. A total of 200 F_3:5_ lines derived from PI 547656 (low KTI) X Glenn (normal KTI) were genotyped using the BARCSoySNP6K_v2 Beadchip. F_3:4_ and F_3:5_ lines were grown in Blacksburg and Orange, Virginia in three years, respectively, and were measured for KTI content using a quantitative HPLC method. We identified three SNP markers tightly linked to the major QTL associated to low KTI in the mapping population. Based on these SNPs, we developed and validated the KASP assays in a set of 93 diverse germplasm accessions. The marker Gm08_44814503 has 86% selection efficiency for the accessions with low KTI and could be used in marker assisted breeding to facilitate the incorporation of low KTI content in soybean seeds.

## 1. Introduction

Soybean (*Glycine max* L. Merr.) is widely recognized as the richest and least expensive source of vegetable protein for livestock, poultry and aquaculture production, which is also used as a source of metabolizable energy (http://soystats.com, date: 18 December 2020). However, soybean seeds have several anti-nutritional factors (ANFs) that affect animal nutrient digestion and absorption, reducing animal growth performance in meat production [1]. The main ANFs in the soybean seeds include proteinase inhibitors, metal chelates, oligosaccharides and antigenic factors. Although the meal industry has established solutions, such as roasting that is applied on raw soybean meal to inactive ANFs, heating can degrade certain essential amino acids, and the feed cost becomes higher due to higher energy cost [2]. Therefore, the most economical and reliable way to improve animals’ ability to digest protein is to feed them with soybean meals containing low concentration of ANFs.

Trypsin inhibitor (TI), one of the ANFs in soybeans, is the major proteinase inhibitor accounting for about 6% of the total protein in soybean seed [3]. TI restrains the activity of a protein-digesting enzyme called trypsin, causing pancreatic hypertrophy/hyperplasia, which ultimately results in an inhibition of animal growth [4]. Trypsin inhibitor in soybean seeds includes two class families, Kunitz TI (KTI, 21 KDa) which is sensitive to thermal treatment and has specificity for trypsin, and Bowman–Birk TI (BBTI, 7–8 KDa), which is more thermo-stable, capable to inhibit trypsin and chymotrypsin [5,6]. Among these two types of TI, KTI serves as the major contributor to the trypsin inhibitor activity in soybeans. KTI is encoded by a gene family containing more than 10 independent genes [7]. Only two KTI genes (KTI3 and GMKTI-1) are sequenced and mapped on chromosome 8 (NCBI.gov, accessed in April 13 2020). KTI3 is the major contributor of the KTI phenotype and is expressed in the soybean seeds [8]. Wang et al. (2011) reported 13 iso-forms of KTI, which are governed by a single gene with multiple alleles [9]. BBTI family in soybean encompass many genes having smaller impacts on the TI phenotype.

Two accessions were originally identified from the USDA’s Germplasm collection: PI 157,440 (‘Kin-du’) and PI 196168, completely lacking the Kunitz trypsin inhibitor protein [10] and both were shown to have a frameshift mutation in the KTI3 gene (*kti*) [7,11]. Despite this mutation, a wide range in trypsin inhibitor activity is observed among germplasm containing either wild type KTI3 or null kti3 allele [12,13] due to a significant proteome rebalancing in KTI mutant lines, resulting in an increased BBTI protein levels [14]. Additionally, both Kunitz and Bowman–Birk trypsin inhibitor classes are influenced by the environment, creating a challenge in breeding selections [12,13,15]. The breeding effort of low TI or TI-free activity seemed very slow [16,17] after ‘Kunitz’ variety, with PI 157,440 as the KTI free donor, was released in the 1970′s, probably because of the establishment of commercial soybean meal processing. Additionally, soybean breeders’ efforts have been focusing on the improvement of productivity characteristics of soybeans such as yield, resistance to disease as well as seed composition such as high protein and high oil concentration. Breeding soybeans for low trypsin inhibitors could potentially replace heat treatment to remove trypsin inhibitor and overcome the problem of reducing nutritional value of soybean meal after heating.

The use of molecular markers for marker-assisted selection (MAS) have been proved to speed up the breeding process and efficiently introgress recessive alleles into crops [18,19,20]. Up to date, previous studies on the development of low KTI soybean cultivars reported the use of SSR markers as tool for breeding selection [21,22,23,24,25]. SSR markers in Chr 08 such as SSR 228, SSR 409 and SSR 429 were reported to be closely linked (0–10 cM) to *ktiti*, the recessive form of *KTi* gene [24]. However, SSRs are generally abundant and polymorphic in non-expressed genomic regions and consequently considered to be selectively neutral. In recent years, SNP markers have started to replace SSRs in population genetic studies as well as in a wide range of other applications [26,27]. SNPs occur in genomes at a much higher frequency than SSRs, but they also occur in intergenic and non-coding regions. However, genome-wide association studies revealed that SNPs located in non-coding regions are often physically linked to functional or regulatory genomic sites, thus reflecting, for example, selection signatures [28]. Moreover, SNP markers are highly stable and can be genotyped in high-throughput systems with a high multiplex ratio such as the PCR-based Competitive Allele Specific technology (KASPar) that is simpler and more cost-effective than other SNP assays. 

In a recent article, Patil et al. (2017) reported a development of a SNP genotyping assay for detection of low KTI in soybean in an F_2_ population of SP6A-206 x PI 542,044 (low KTI). However, Patil et al. did not report what phenotyping system they used to discriminate the low KTI lines from the high ones, making not clear if the KASP clustering correspond to true low and high KTI phenotypes. Moreover, we tested the reported SNP genotyping assay in the Glenn (high KTI) x PI 547,656 (low KTI) population with no successful results, perhaps because PI 542,044 and PI 547,656 do not have common low KTI genes. In 2018, Rosso et al. developed an HPLC method as a reliable method to quantify KTI in soybean seeds. showing that this quantification method was strongly correlated with two popular activity assays for measuring total TI activity. Additionally, the HPLC method overcomes the flaws of activity assays because it can detect low levels of KTI on nonfunctional mutated soybean lines, which is impossible for activity assays, and can measure only KTI, not the mixture of KTI and BBTI as activity assays do. Therefore, the objectives of this study were: (i) to identify SNP markers associated with the QTL that confers low KTI in soybean seeds; and (ii) to develop and validate a breeder -friendly KASP SNP genotyping assay linked to low KTI content derived from ‘Kin-du’ as the KTI free donor, that could be used in marker-assisted selection to incorporate the low KTI trait into elite breeding lines.

## 2. Results

### 2.1. Kunitz Trypsin Inhibitor Phenotyping

**Figure 1:**
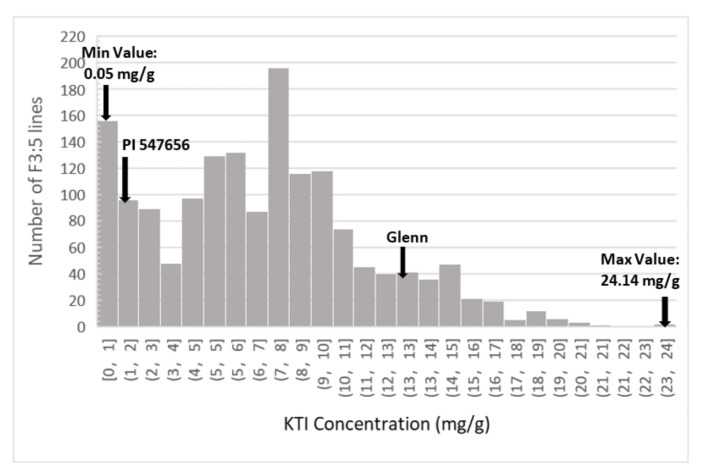
Frequency distribution of KTI concentration (mg/g) of in 200 F_3:5_ lines developed from a cross between Glenn (normal KTI) and PI 547,656 (low KTI) planted in Blacksburg and Orange, VA in 2016 and 2017.

**Figure 2:**
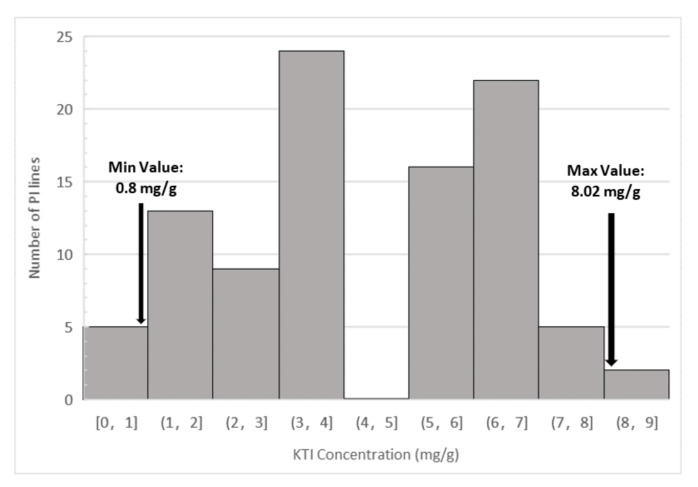
Frequency distribution of KTI concentration (mg/g) of Plant Introductions (PIs) selected for the SNP Validation.

The HPLC method successfully quantified the concentration of KTI in soybean seeds from the two selected parents, F_3:4_ (data not shown) and F_3:5_ lines from the mapping population and the 93 PIs selected for the SNP validation. Analysis of variance and least squares means Student’s *t*-test showed there was a significant (*p* = 0.007) variation in KTI concentration between the two parents, Glenn and PI 547,656 (Table 1 and Table 2). There was no significant difference between location and the parent by location interaction was not significant either (Table 1 and Table 2). Analysis of variance indicated that there was a significant difference among the F_3:5_ lines (Table 3) on KTI concentration, ranging from 0.05 mg/g to 24.14 mg/g (standard errors = 0.12) (Figure 1). The non-parametric Kruskal–Wallis test and analysis of variance showed that there was a statistically significance difference in KTI concentration between the different locations, Blacksburg and Orange (*x^2^ =* 3.78, *p* = 0.05) and also between years (*x^2^ =* 164.76, *p* < 0.0001) (Table 3 and Table 4). Frequency distribution of the 93 PIs selected for SNP validation also showed a wide range of KTI concentration, ranging from 0.8 mg/g to 8.02 mg/g (Figure 2).

The broad-sense heritability (*H*^2^) of KTI was estimated to be 54% and was calculated using variance components for KTI of the mapping population across two years (2016 and 2017) and two locations (Blacksburg and Orange, VA, USA).

### 2.2. QTL Analysis

**Figure 3:**
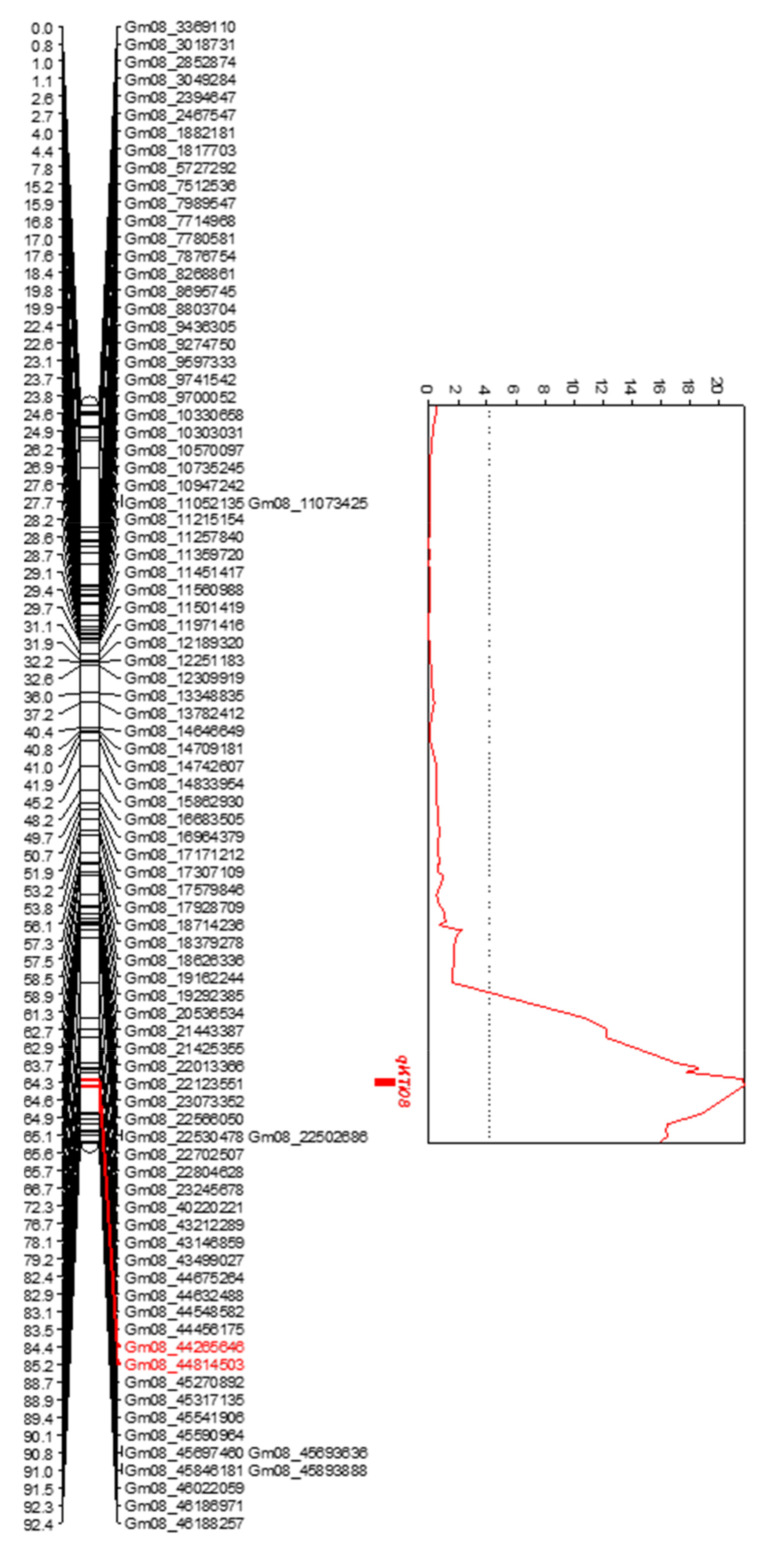
Quantitative trait loci (QTL) mapping of *qKTI08* in chromosome 8 (LG A2) in the biparental mapping population derived from Glenn x PI 547656.

Out of 6000 evaluated SNPs, a total of 1357 polymorphic SNPs (22.6%) were selected for establishing the genetic linkage map. The linkage map generated by SMA from the mapping population consisted of 20 chromosomes, which spanned 1278.69 cM and were defined by 1272 SNP markers. The constructed map had a coverage ranging from 0.097 to 0.312 cM with an average coverage of 0.215 cM (Table 5). One QTL conferring low KTI concentration was identified using IciMapping analysis. The QTL located in chromosome 8 (LG A2) was mapped between SNPs Gm08_44814503 and Gm08_45270892, with a peak close to Gm08_44814503 (Figure 3). QTL detected in 2016 and 2017 showed LOD score of 21.93 and 23.44 and explained 39.95 and 39.83% of the total variation of the trait, respectively (Table 6). This QTL is stable across years and significantly associated with quantities of KTI. We tentatively named this QTL for KTI concentrations as *qKTI08*. The additive effect (Add) of *qKTI08* was −1.88 mg/g in 2016 and −3.99 mg/g in 2017, indicating that the allele at this QTL inherited from the low KTI parent (PI 547656) reduced KTI content (Table 6). When analyzing the average KTI content across 2016 and 2017, *qKTI08* showed a LOD score of 24.95, explained 41.34% of total phenotypic variation, and had a −2.93 mg/g additive affect (Table 6).

The SNPs Gm08_44814503 and Gm08_45270892 were located at 85.22 and 88.74 cM on chromosome 8 (LG A2), respectively, in the most recently developed high density linkage map for soybeans [29]. Based on the physical position information of the SNPs, the physical distance between these markers is 1.91 Mb; and the corresponding genome positions of Gm08_44814503 and Gm08_45270892 in the reference soybean genome (Glyma.Wm82.a2.V1 assembly) are 35.14 Mb–37.05 Mb [29].

### 2.3. SNP Validation

**Figure 4:**
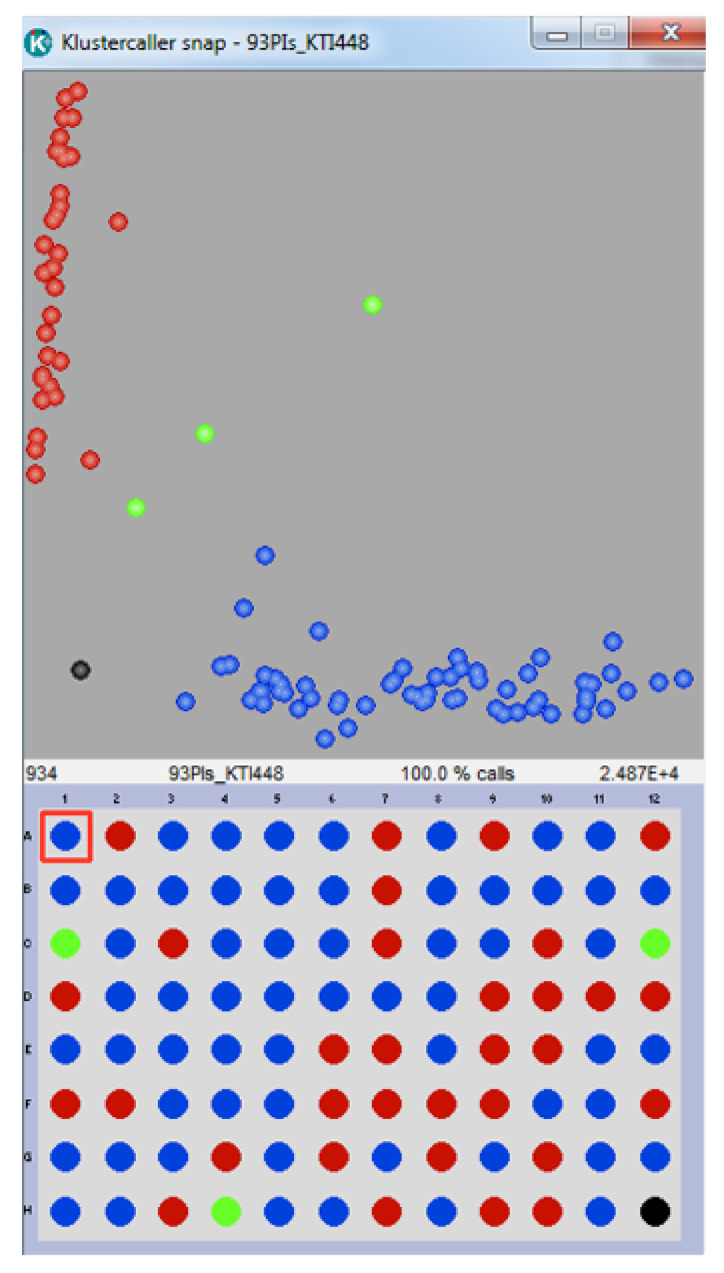
KlusterCaller^TM^ genotyping results. Gm08_44814503 SNP genotyping results of the 93 PIs and Glenn (well 12F) and PI 547,656 (well 12G) are shown in the Cartesian cluster plot. Blue data points are homozygous lines for the low KTI allele reported by FAM dye, green data points are heterozygous lines and red data points are homozygous lines for the normal KTI allele reported as HEX dye. The black data point represents the no template control (NTC). The DNA sample 96-well plate layout is shown below the cluster plot.

Molecular markers flanking the *qKTI08* region on chromosome 8 that were significantly associated (*p*-value < 0.0001) with low Kunitz trypsin inhibitor content across years and locations were identified using single marker analysis (SMA) on IciMapping. Table 7 shows five SNPs significantly associated with KTI content in the mapping population in 2016 and 2017. All these SNPs are tightly linked to *qKTI08* (Figure 2). We converted three of them to KASP assays to validate the robustness of these SNP assays on selected 93 plant introductions with low and high KTI content. Out of the 93 PIs phenotyped for KIT concentration by HPLC analysis, 51 lines were normal KTI and 42 were low KTI. Table 2 shows the selection efficiency of these three SNP markers. The three SNP markers, Gm08_44265646, Gm08_44814503 and Gm08_45317135, were polymorphic between the PIs and have a selection efficiency of 64%, 86% and 31%, respectively. Out of a total of 42 PIs with low KTI phenotype, 15 (36%) and 29 (69%) low KTI PIs were not selected by Gm08_44265646 and Gm08_45317135, respectively. However, only six (14%) low KTI PIs were not selected by Gm08_44814503 SNP marker, which means that Gm08_44814503 SNP marker was able to select 86% of the low KTI PIs from the core collection (Table 2). Figure 4 shows the results of the cluster analysis of Gm08_44814503 SNP marker where 31 lines showed the normal KTI genotype, three lines showed the heterozygous genotype and 59 lines showed the low KTI genotype, the last two samples in the graph belong to Glenn (normal KTI) and PI 547,656 (low KTI).

## 3. Discussion

**Figure 5:**
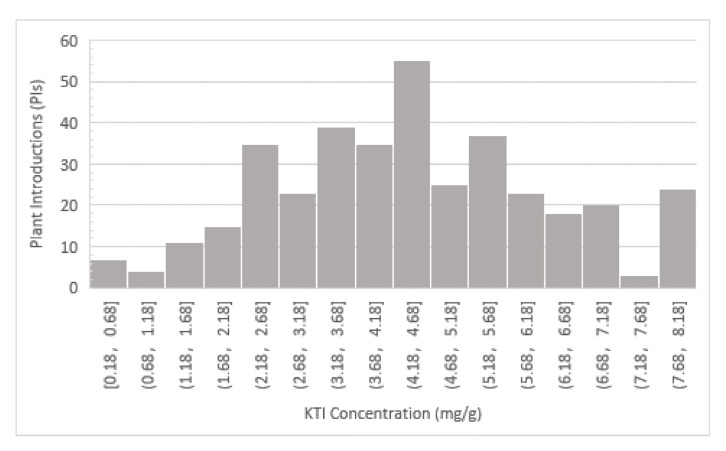
Frequency distribution of KTI concentration (mg/g) of 500 soybean accessions collected by the National Genetic Resources Program (USDA Soybean Germplasm Collection).

The presence of Kunitz trypsin inhibitor (KTI) in soybean seeds has a negative impact on the feed and food industry since it affects animal nutrient digestion and absorption, reducing animal growth performance in meat production [4]. The best cost-efficient way to overcome this problem would be to use soybean cultivars with low or null KTI content. In an effort to develop soybean cultivars with low KTI content, we crossed the soybean cultivar Glenn (normal KTI content of 12.63 mg/g) with the low KTI PI 547,656 (1.38 mg/g) that carries the low KTI allele in 2014. The biparental population of 200 F_3:5_ lines presented a wide range of KTI content (0.5–24.14 mg/g) (Figure 5). We estimated that *H^2^* for KTI content as 0.54, which is considered a medium heritability [30]. A moderate broad-sense heritability and significant difference of KTI content between years and locations (Table 3 and Table 4) showed that genetic and environment affect this trait. Vollmann et al. (2003) also reported that KTI activity in soybean was significantly affected by the environment and genotype. Thus, the use of molecular markers would play an important role to incorporate the low KTI trait into elite breeding lines. Molecular marker-based selection paired with phenotypic selection is an efficient way to advance breeding lines with the desirable trait, plus selective elimination of undesirable traits, in the shortest possible time. Microsatellite markers, such as Satt 228, Satt 409 and Satt 429 have been previously used when breeding for low or null KTI content [24,31,32], however, single nucleotide polymorphism markers have gained importance in the breeding programs due to their large amount on the genome, high cost efficiency, and high repeatability.

This study confirms the presence of a QTL on Chromosome 8 (LG A2) associated with low concentration of KTI previously reported. In this study, one QTL (*qKTI08*) associated with KTI was detected in PI 547,656 on chromosome 8 with a confidence interval from Gm08: 44,814,503 to Gm08:45270892 (Figure 2). This QTL is desired for marker-assisted selection due to its stability across years and locations. As today, there are five published genes in this region, two calcineurin *B*-like protein, two heat shock cognate protein and one proliferating cell nuclear antigen [33]. However, close to this region, between Gm08_45730511 and Gm08_45786029 SNP markers, seven Kunitz trypsin inhibitor proteins have been identified, including KTI3 [34,35]. Therefore, chromosome 8 harbors several genes associated with Kunitz trypsin inhibitors. Moreover, previously published SSR markers used for breeding low TI lines [24,25,31,32] are also found close to this QTL, between region Gm08:46124977 to Gm08:47218108.

We found five SNP markers closely associated (*p* < 0.0001) with low KTI content across years and locations (Table 7). Three out of these five SNP markers were converted to KASP assays and use for validation on the genetically diverse 93 PIs. Gm08_44814503 KASP SNP assay was able to select 86% of the low KTI PIs from the validation panel. This SNP marker will be more useful in MAS than previously developed assay by Patil et al., 2017, since our was validated in 93 plant introductions that have much more diverse genetic background than Kin-du alone as the low KTI donor. The SNP assay previously developed by Patil et al., 2017 for low KTI was based on a mutation on PI 157,740 [7], however they only tested this assay on an F_2_ population derived from SP6A-209 x PI 542,044 lines, where PI 542,044 is a germplasm with null KTI. The validated KASP assay proposed in this work offers a rapid and more economical genotyping assay compared to previous assays [36,37] in practical applications such as MAS backcrossing, advance breeding lines, and germplasm identification. Moreover, KASP assays has been proven to work very well for many traits in the soybean genome [38].

Current published TI activity assays can only test total TI activity, but cannot differentiate KTI or BBTI activity. Therefore, we focused on KTI content in this study. However, breeding only for low or null KTI content in soybean would not be sufficient for practical applications for a few reasons, such as the effect of environmental conditions on the trait, the interaction of environment and genotype, the expression of one of the 13 iso-forms of *KTI3* gene in the seeds and presence of Bowman–Birk TI affect the TI activity in soybean seeds. Gillman et al. indicated that low KTI lines had dramatically increased Bowman–Birk TI level [14]. Therefore, the practical and effective approach for expanding the soybean meal value for the poultry industry is to reduce both KTI and BBTI content in soybean. The implementation of low total TI into breeding lines would reduce the need of postharvest heat treatment, which will bring benefits to the soybean feed and food processing industry.

In conclusion, this study successfully mapped the QTL on chromosome 8 linked to KTI content that was previously published and developed a robust, cost-effective and high-throughput SNP assay that could be applied across different genetic backgrounds in soybean breeding programs to facilitate marker-assisted selection for low KTI content in soybean seeds with great efficiency and accuracy. The KASPar KTI assay is a breeder-friendly SNP detection system for selection of low KTI soybean lines since KASPar assay samples are amplified with a standard thermal cycler and can be genotyped with any type of FRET reader, which can easily be done by many breeding programs. Future work will focus on the identification of QTLs and SNP markers to select soybean breeding lines low in BBTI content as well, as *double null* will have a stronger application to the feed and food industry.

## 4. Methods

### 4.1. Population Development

Parental soybean lines were chosen based on the amount of KTI in the seeds. Glenn, developed and released by Virginia Tech in 2009, was chosen as a normal KTI concentration parent. PI 547,656 (cultivar name: L81-4871) was chosen as a low KTI parent. PI 547656, derived from a cross ‘Clark 63′ x PI 157,440 (‘Kin-du’), was developed by the USDA-ARS and the Illinois Agricultural Experimental Station in 1986 [15]. Glenn X PI 547,656 crosses were made in summer of 2014 at the Virginia Tech farm in Blacksburg, VA. F_1_ plants were spaced-planted and increased for two generations with modified pod descent at each generation in a Puerto Rico winter nursery in winter of 2014. Six SSR markers (Satt449, Satt197, Satt281, Satt268, Satt431 and Satt345), which were polymorphic between parents, were used to verify true hybrids. A total of 200 individuals of the F_2_ were advanced to F_3_ in 2015. The parents and F_2:3_ lines were separately planted in single, 3.05 m long rows with 0.76 m row spacing and about 30 seeds per meter arranged in a randomized complete block design in Blacksburg, VA, USA in 2015. Two parents and a population of F_3:4_ lines were grown in Blacksburg and Orange, VA, USA in 2016, and two parents and a population of F_3:5_ lines were grown in the same two locations in 2017 using the same experimental design. 

### 4.2. Kunitz Trypsin Inhibitor Phenotyping

The two parents and 200 progenies of the F_3:4_ and F_3:5_ mapping population were phenotyped for KTI content using a quantitative HPLC method [39]. F_3:4_ phenotypic data are not shown. Briefly, 10 mg of finely grounded soybean seed powder was mixed with 1.5 mL of 0.1 M sodium acetate buffer, pH 4.5. Samples were vortexed and shaken for 1 h at room temperature. The samples were centrifuged at 12,000 rpm for 15 min. One-mL of the supernatants were filtered through a syringe with an IC Millex-LG 13 mm mounted 0.2 µm low protein binding hydrophilic millipore (PTFE) membrane filter (Millipore Ireland BV, Carrigtwohill, Republic of Ireland). The Kunitz trypsin inhibitor in solution were separated on an HPLC Agilent 1260 Infinity series (Agilent Technologies, Santa Clara, CA, USA), equipped with a guard column (4.6 × 5 mm^2^) packed with POROS^®^ R2 10 µm Self Pack^®^ Media and Poros R2/H perfusion analytical column (2.1 × 100 mm^2^, 10 µm). The mobile phase A consisted of 0.01% (*v*/*v*) trifluoroacetic acid in Milli-Q water and mobile phase B was 0.085% (*v*/*v*) trifluoroacetic acid in acetonitrile. The injection volume was 10 µL and the detection wavelength was 220 nm. The final KTI concentration of each sample was calculated using the initial weight of each sample to calculate the KTI concentration on a dry weight basis with results reported as milligrams per gram (mg/g).

### 4.3. DNA Extraction

A bulk sample of young leaves from three individuals of each F_3:5_ lines of the mapping population and each PI from the validation panel were collected and stored at −80 °C until DNA extraction. Leaves were freeze dried using a FreeZone 6 Liter Console Freeze Dry System (−56 °C and 0.220 mbar) and about 200 mg of leaf tissue were placed in a 2.5 mL tube. Tissue was ground to fine powder in liquid N_2_ using glass stirring rods. Total genomic DNA was isolated from leaf tissue (0.20 g) by a modified protocol from the CTAB method of [40].

### 4.4. Single-Nucleotide Polymorphism Genotyping

Fifty nanograms of genomic DNA for the parental lines and the population of F_3:5_ lines were sent to USDA–ARS Soybean Genomics and Improvement Laboratory (Beltsville, MD, USA) and genotyped using the Illumina BARCSoySNP6K_v2 Beadchip containing 6000 SNPs. Single-nucleotide polymorphism genotyping was conducted according to Song et al. 2013 [41] on the Illumina platform following the Infinium HD Assay Ultra Protocol (Illumina, Inc., San Diego, CA, USA). Single-nucleotide polymorphism allele calling was done using the Genome Studio Module v2.0.3 software (Illumina, Inc.). The low KTI parent was scored as A and high KTI parent was scored as B. SNPs with no call and the monomorphic SNPs between parents were discarded. SNPs with low minor allele frequency (MAF) (<10%) and high missing data ratio (<5%), as well as severe segregation distortion were filtered for quality control.

### 4.5. Linkage Map Construction and QTL Analysis

Linkage maps were constructed by JoinMap v4 [42] using a regression approach with a minimum logarithm of odds (LOD) threshold of 3 for linkage group construction. Recombination frequencies were converted to centimorgan (cM) using Kosambi’s mapping function [43]. QTL analysis was performed by single marker analysis, interval mapping and composite interval mapping implemented in IciMapping v 4.1 [44]. For single marker analysis, SMA, *p* < 0.0001 was used as a threshold for significant markers. In the composite interval mapping (CIM) and simple interval mapping (SIM), the empirical significance threshold was determined by 1000-time permutation with a walk speed of 1 cM and significance level of 0.05. MapChart [45] was used to create the LOD plots based on JoinMap v4 and IciMapping v4.1 data.

### 4.6. KASP Marker Development

The SNPs tightly linked to major QTL identified in the mapping population (Table 7) were converted into Kompetitive Allele Specific PCR (KASP) SNP genotyping assays (LGC, Middlesex, UK) with the flanking sequences obtained from the *G. max* genome Glyma.Wm82.a1 (Schmutz et al. 2010) following Zhu et al. [46]. Briefly, the KASP oligos were synthesized by IDT (IDT, Iowa, USA), with primers carrying FAM tail (5′-GAAGGTGACCAAGTTCATGCT-3′) or VIC tail (5′-GAAGGTCGGAGTCAACGGATT-3′), and the target SNP in the 3′ end. Primer mix and PCR reaction was set up following LGC Genomics recommendation (46 µL distilled water, 30 µL common primer [100 µM], and 12 µL of each tailed primer [100 µM]) [46]. Thermocycling conditions consisted of the initial hot-start step at 95 °C for 15 min., followed by 10 cycles of touchdown PCR (annealing 65 °C to 57 °C, decreasing 0.8 °C per cycle), then 35 cycles of 20 s at 94 °C and 60 s at 57 °C. PCR and fluorescent endpoint reading were performed in FLUOstar Omega microplate reader (BMG LABTECH) [46].

### 4.7. KASP Marker Validation

The 6749 MGs IV and V soybean accessions collected by the National Genetic Resources Program (USDA Soybean Germplasm Collection) were arbitrarily grouped in 500 clusters based on their genetic distance which were calculated using 42,509 SNPs that were included in the SoySNP50K [47]. One accession with the highest average genetic distance within each cluster was selected to form a collection with 500 accessions. These 500 accessions were planted in single, 1.83 m long rows with 0.15 m row spacing and about 30 seeds per meter arranged in a complete randomized design with no replication in Puerto Rico in the winter of 2016, and in a randomized complete block design with three replications in Blacksburg, VA, USA and Clayton, NC, USA in 2017. The 500 accessions were phenotyped for KTI content by HPLC analysis (Figure 5) and 93 diverse plant introductions (PIs) were selected based on KTI content to have a wide range of KTI concentration for the SNP validation. Out of these 93 PIs, 42 were low KTI and 51 were normal KTI. We selected 93 PIs to fill a 96-well plate for PCR amplification (93 PIs, plus Glenn as a negative control, PI 547,656 as a positive control and one1 blank well). DNA was extracted from bulk leaves of 6–8 plants of each of the selected 93 diverse PIs for the SNP validation. DNA was extracted following the same procedure as used for the mapping population. Selection efficiency (SE) of the selected KASP markers linked to low KTI was calculated as follows: SE = total number of marker-selected lines with low KTI phenotype ÷ total number of lines with low KTI phenotype x 100 (Table 8).

### 4.8. Statistical Data Analysis

Concentration of KTI, collected in 2016 and 2017, were analyzed using JMP statistical version Pro 14.2.0 (SAS Institute). KTI concentration was analyzed by analysis of variance and the non-parametric method Kruskal–Wallis test for the 2016 and 2017 data at two locations, Blacksburg and Orange, VA, USA. Student’s t test was used to determine if mean of KTI concentration of parents and lines differed significantly at *p* = 0.05. Histograms of KTI concertation were also performed by JMP. Analysis of variance was used to determine phenotypic differences between parents, Glenn and PI 547656. Variance-component heritability estimates were calculated by analyses of variance using R software. Broad-sense heritability of seed coat deficiency was estimated using the equation:

*H*^2^ = s^2^_g_/[s^2^_g_ + (s^2^_ge_/e) + (s^2^/re)]

where *H*^2^ is heritability, s^2^_g_ is genotypic variance, s^2^_ge_/e is genotype x environment interaction variance, s^2^ is error variance, r is the number of replications, and e is the number of environments [48].

## Figures and Tables

**Table 1:** Analysis of variance of Kunitz trypsin inhibitor concentration (KTI) between the two parents Glenn (normal KTI) and PI 547,656 (low KTI) planted in Blacksburg and Orange, VA in 2016 and 2017.

Effect	*df*	Sum of Squares	*f*-Value	*p*-Value
Environments	3	63.36	1.33	0.27
Residual Error	14	1131.39		
Parent	1	505.80	10.50	0.017
Residual Error	14	688.95		
Parent by Location interaction	3	50.83	10.60	0.32

**Table 2:** Least squares mean differences using Student’s t test of Kunitz trypsin inhibitor concentration (KTI) between the two parents Glenn (normal KTI) and PI 547,656 (low KTI) planted in Blacksburg and Orange, VA in 2016 and 2017.

Level	Student’s *t* Test Grouping	Least Square Mean	*α ^a^*	*t*-Value
Glenn	A	12.63	0.05	2.18
PI 547656	B	1.38		
Blacksburg	A	8.34	0.05	1.96
Orange	A	8.06		

Levels not connected by same letter are significantly different. ^a^ level of significance α = 0.05.

**Table 3:** Analysis of variance of Kunitz trypsin inhibitor concentration (KTI) among 200 F_3:4_ lines (2016) and 200 F_3:5_ lines (2017) as well as parents Glenn (normal KTI) and PI 547,656 (low KTI) planted in Blacksburg and Orange, VA.

Effect	*df*	Sum of Squares	*f*-Value	*p*-Value
Genotype	201	18446.89	14.81	<0.0001
Residual Error	1614	12333.51		
Year	1	3644.435	588.13	<0.0001
Residual Error	1614	27135.97		
Location	1	67.21	10.84	0.0001
Residual Error	1614	30713.18		
Replication	1	12.86	2.08	0.15
Genotype by Location interaction	201	1111.05	0.89	0.82

**Table 4:** Kruskal–Wallis test of Kunitz trypsin inhibitor concentration (KTI) among 200 F_3:4_ lines (2016) planted in Blacksburg and Orange, VA in 2016 and 2017.

Level	Count	Score Sum	Expected Score	Score Mean
Location				
Blacksburg	800	622434	640400	778.04
Orange	800	658366	640400	822.95
1-way Test				
Chi-square	*df*	*Prob > ChiSq*		
3.78	1	0.05		
Year				
2016	800	521809	640400	652.26
2017	800	758991	640400	948.73
1-way Test				
Chi-square	*df*	*Prob > ChiSq*		
164.76	1	<0.0001		

level of significance α = 0.05.

**Table 5:** Summary of single nucleotide polymorphism (SNP) marker mapping in F3-derived F5 mapping population from Glenn x PI 547656.

Chr ^a^	LG ^b^	Length (cM) ^c^	No. all SNPs ^d^	All SNP coverage ^e^	No. P-SNPs ^d^	P-SNPs coverage ^e^	No. of mapped SNPs
1	D1a	70.90	263	0.270	71	0.999	71
2	D1b	88.42	329	0.269	80	1.105	79
3	N	50.14	280	0.179	86	0.583	72
4	C1	38.42	266	0.144	64	0.600	50
5	A1	87.63	294	0.298	95	0.922	95
6	C2	95.03	313	0.304	86	1.105	86
7	M	55.13	319	0.173	45	1.225	45
8	A2	92.40	380	0.243	91	1.015	90
9	K	81.39	261	0.312	77	1.057	75
10	O	66.34	317	0.209	77	0.862	72
11	B1	63.03	279	0.226	39	1.616	39
12	H	68.79	258	0.267	45	1.529	44
13	F	70.78	381	0.186	61	1.160	60
14	B2	27.58	275	0.100	66	0.418	38
15	E	65.48	305	0.215	71	0.922	70
16	J	45.66	238	0.192	54	0.846	54
17	D2	55.85	265	0.211	41	1.362	40
18	G	37.01	380	0.097	107	0.346	92
19	L	40.36	326	0.124	41	0.984	40
20	I	78.35	271	0.289	60	1.306	60
Mean		63.93	300	0.215	67.85	0.998	63.60
Total		1278.69	6000		1357		1272

^a^ Chromosome. ^b^ Linkage group. ^c^ Chromosome length in centimorgan. ^d^ SNP, single nucleotide polymorphism. Number of markers screened for each chromosome: all SNPs (all 6K SNPs), and P-SNPs (polymorphic SNPs). ^e^ Distribution of SNP markers on chromosomes (total chromosome length/number of SNP markers screened): all SNPs (all 6K SNPs), and P-SNPs (polymorphic SNPs).

**Table 6:** Quantitative trait loci (QTL) mapping, marker intervals, logarithm of odds (LOD) scores, percentage of variance (PVE), and additive effects of QTL for KTI concentration (qKTI08) in the F3:5 mapping population derived from Glenn x PI 547656.

Year	QTL Name	Chr. (LG) ^a^	Position (cM)	Confidence Interval ^b^	Peak	LOD^c^	PVE (%) ^d^	Add (mg/g) ^e^
2016	*qKTI08*	8 (A2)	86	Gm08_44814503	Gm08_44814503	21.93	39.95	−1.88
				Gm08_45270892				
2017	*qKTI08*	8 (A2)	85	Gm08_44814503	Gm08_44814503	23.44	39.83	−3.99
				Gm08_45270892				
Avg 2016–2017	*qKTI08*	8 (A2)	86	Gm08_44814503	Gm08_44814503	24.95	41.94	−2.93
				Gm08_45270892				

^a^ Chromosome (Linkage Group). ^b^ Physical position of interval markers in base pairs on the William 82 reference genome (Wm82.a2.v1). ^c^ Logarithm of odds. ^d^ Percentage of variance explained. ^e^ Additivity effect (mg/g) on the specific QTL (a negative effect indicated a low concentration of KTI contributed by PI 547656).

**Table 7:** KASP assay primers sequences significantly associated (*p* < 0.0001) with concentration of KTI in the mapping population derived from Glenn x PI 547656.

Chr. (LG) ^a^	Marker ID	Position (cM)	Sequences	Low KTI Allele ^b^	Will82 Allele ^c^	LOD ^d^
8 (A2)	Gm08_44265646_C_T	84.42	FAM_primer: GAAGGTGACCAAGTTCATGCCTTATGCACGCGCCGAAACTTA	T	C	21.68 ***
			VIC_primer: GAAGGTCGGAGTCAACGGATTCTTATGCACGCGCCGAAACTTG			
			Common reverse primer: TGTACTCACCTGAAACTTCC			
8 (A2)	Gm08_44814503_C_T	85.22	FAM_primer: GAAGGTGACCAAGTTCATGCCTGGAGGTGTTGGCATTGAGGA	T	C	21.73 ***
			VIC_primer: GAAGGTCGGAGTCAACGGATTCTGGAGGTGTTGGCATTGAGGG			
			Common reverse primer: TCGACATGATTCTTTTAGAC			
8 (A2)	Gm08_45270892_A_G	88.73	FAM_primer: GAAGGTGACCAAGTTCATGCTAATGACAAAGGATAGATTGC	G	A	18.84 ***
			VIC_primer: GAAGGTCGGAGTCAACGGATTTAATGACAAAGGATAGATTGT			
			Common reverse primer: TGAACAAATATATATTAAGCC			
8 (A2)	Gm08_45317135_T_G	88.90	FAM_primer: GAAGGTGACCAAGTTCATGCACAGAAGAACAACCCGGCACC	G	T	18.53 ***
			VIC_primer: GAAGGTCGGAGTCAACGGATTACAGAAGAACAACCCGGCACA			
			Common reverse primer: CTTGGTTTCCGATGCTGTATC			
8 (A2)	Gm08_45541906_A_C	89.43	FAM_primer: GAAGGTGACCAAGTTCATGCGTCTTCTGATTGCTGAAGCAGG	C	A	17.6 ***
			VIC_primer: GAAGGTCGGAGTCAACGGATTGTCTTCTGATTGCTGAAGCAGT			
			Common reverse primer: AAATAAAACATCTAATTTTTA			

^a^ Chromosome (Linkage Group). ^b^ Allele linked to low concentration of KTI. ^c^ Allele linked to high concentration of KTI. ^d^ Logarithm of odds, *** SNPs significantly associated with low KTI concentration at a *p*-value < 0.0001.

**Table 8:** Selection efficiency of the SNP assays in 93 Plant Introductions (PIs) collected by the National Genetic Resources Program to form a MGs IV and V core collection.

			Genotypic Class (*n*)	
Phenotype	Number of Accessions (n)	Gm08_44265646	Gm08_44814503	Gm08_45317135
Normal KTI (mg/g) ^a^	51	48	31	64
Heterozygous	.	0	3	6
Low KTI (mg/g)	42	45	59	23
No. low KTI lines selected by marker		27	36	13
No. of low KTI lines not selected by the marker		15	6	29
Marker selection Efficiency ^b^		64%	86%	31%

^a^ Normal KTI phenotypes consist of wild type and heterozygous phenotypes. ^b^ Marker selection Efficiency: Total number of marker-selected lines with low KTI phenotype ÷ total number of lines with low KTI phenotype x 100.

## Data Availability

Not applicable References.

## References

[1] Yen J.T., Jensen A.H., Simon J. (1977). Effect of Dietary Raw Soybean and Soybean Trypsin Inhibitor on Trypsin and Chymotrypsin Activities in the Pancreas and in Small Intestinal Juice of Growing Swine. J. Nutr..

[2] Boge E.L., Boylston T.D., A Wilson L. (2009). Effect of cultivar and roasting method on composition of roasted soybeans. J. Sci. Food Agric..

[3] Hymowitz T., Friedman M. (1986). Genetics and Breeding of Soybeans Lacking the Kunitz Trypsin Inhibitor. Nutritional and Toxicological Significance of Enzyme Inhibitors in Foods.

[4] Liener I.E. (1994). Implications of antinutritional components in soybean foods. Crit. Rev. Food Sci. Nutr..

[5] Chen Y., Xu Z., Zhang C., Kong X., Hua Y. (2014). Heat-Induced Inac-tivation Mechanisms of Kunitz Trypsin Inhibitor and Bowman-Birk Inhibitor in Soymilk Pro-cessing. Food Chem..

[6] Galão O., Carrão-Panizzi M., Mandarino J., Leite R., Claus T., Visentainer J. (2014). Kunitz Trypsin Inhibitor and Phytic Acid Levels in Conventional and Genetically Modified Soy-bean Seeds from Londrina and Ponta Grossa, South Brazil. Acta Sci. Technol..

[7] Jofuku K.D., Goldberg R.B. (1989). Kunitz Trypsin Inhibitor Genes Are Differentially Ex-pressed During the Soybean Life Cycle and in Transformed Tobacco Plants. Plant Cell.

[8] De Moraes R.M.A., Soares T.C.B., Colombo L.R., Salla M.F.S., Barros J.G.D.A., Piovesan N.D., De Barros E.G., Moreira M.A. (2006). Assisted Selection by Specific DNA Markers for Genetic Elimination of the Kunitz Trypsin Inhibitor and Lectin in Soybean Seeds. Euphytica.

[9] Wang K.-J., Li X.-H., Yamashita T., Takahata Y. (2011). Single Nucleotide Mu-tation Leading to an Amino Acid Substitution in the Variant Tik Soybean Kunitz Trypsin Inhibitor (Skti) Identified in Chinese Wild Soybean (Glycine Soja Sieb. & Zucc.). Plant Syst. Evol..

[10] Orf J.H., Hymowitz T. (1979). Inheritance of the Absence of the Kunitz Trypsin Inhibitor in Seed Protein of Soybeans 1. Crop. Sci..

[11] Krishnan H.B. (2001). Characterization of a soybean [Glycine max (L.) Merr.] mutant with reduced levels of Kunitz trypsin inhibitor. Plant Sci..

[12] Marchetti S., Giordano A., Chiabå C. (1995). Within-Plot and within-Plant Varia-tion for Seed Content of Soya Bean Protease Inhibitors. J. Sci. Food Agric..

[13] Vollmann J., Grausgruber H., Wagentristl H., Wohleser H., Michele P. (2003). Trypsin inhibitor activity of soybean as affected by genotype and fertilisation. J. Sci. Food Agric..

[14] Gillman J.D., Kim W.-S., Krishnan H.B. (2015). Identification of a New Soybean Kunitz Trypsin Inhibitor Mutation and Its Effect on Bowman−Birk Protease Inhibitor Content in Soybean Seed. J. Agric. Food Chem..

[15] Bernard R.L., Hymowitz T. (1986). Registration of L81-4590, L81-4871, and L83-4387 Soybean Germplasm Lines Lacking the Kunitz Trypsin Inhibitor. Crop Sci..

[16] Peric V., Srebric M., Dragicevic V., Nikolic A., Mikic A., Drinic S.M. (2014). Development of Soybean Varieties with Specific Nutritional Composition of Grain. J. Hyg. Eng. Des..

[17] Schmidt M.A., Hymowitz T., Herman E.M. (2015). Breeding and characterization of soybeanTriple Null;a stack of recessive alleles of Kunitz Trypsin Inhibitor, Soybean Agglutinin, and P34 allergen nulls. Plant Breed..

[18] Saghai Maroof M.A., Jeong S.C., Gunduz I., Tucker D.M., Buss G.R., Tolin S.A. (2008). Pyramid-ing of Soybean Mosaic Virus Resistance Genes by Marker-Assisted Selection. Crop Sci..

[19] Rajpurohit D., Kumar R., Kumar M., Paul P., Awasthi A., Ba-sha P.O., Puri A., Jhang T., Singh K., Dhaliwal H.S. (2011). Pyramiding of Two Bacterial Blight Resistance and a Semidwarfing Gene in Type 3 Basmati Using Marker-Assisted Selection. Euphytica.

[20] Howell P., Leigh F., Bates R., Gosman N., Trafford K., Powell W., Smith A.M., Greenland A. (2013). Rapid marker-assisted development of advanced recombinant lines from barley starch mutants. Mol. Breed..

[21] Kumar V., Rani A., Shukla S., Jha P. (2020). Development of Kunitz Trypsin inhibitor free vegetable soybean genotypes through marker-assisted selection. Int. J. Veg. Sci..

[22] Rani A., Kumar V., Shukla S., Jha P., Tayalkar T., Mittal P. (2020). Changes in storage protein composition on genetic removal of Kunitz trypsin inhibitor maintain protein content in soybean (*Glycine max*). J. Agric. Food Res..

[23] Kumar V., Rani A., Rawal R. (2013). Deployment of Gene Specific Marker in Develop-ment of Kunitz Trypsin Inhibitor Free Soybean Genotypes. Indian J. Exp. Biol..

[24] Kim M.S., Park M.J., Jeong W.H., Nam K.C., Chung J.I. (2006). SSR marker tightly linked to the Ti locus in Soybean [*Glycine max* (L.) Merr.]. Euphytica.

[25] Rani A., Kumar V., Mourya V., Singh R.K., Husain S.M. (2011). Validation of SSR markers linked to null kunitz tryspin inhibitor allele in Indian soybean [*Glycine max* (L.) Merr.] population. J. Plant Biochem. Biotechnol..

[26] Brumfield R.T., Beerli P., Nickerson D.A., Edwards S.V. (2003). The Utility of Sin-gle Nucleotide Polymorphisms in Inferences of Population History. Trends Ecol. Evol..

[27] Guichoux E., Lagache L., Wagner S., Chaumeil P., Léger P., Lepais O., Lepoittevin C., Malausa T., Revardel E., Salin F. (2011). Current trends in microsatellite genotyping. Mol. Ecol. Resour..

[28] Kim S., Plagnol V., Hu T.T., Toomajian C., Clark R.M., Ossowski S., Ecker J.R., Weigel D., Nordborg M. (2007). Recombination and Linkage Dise-quilibrium in Arabidopsis Thaliana. Nat. Genet..

[29] Song Q., Jenkins J., Jia G., Hyten D.L., Pantalone V., Jackson S.A., Schmutz J., Cregan P.B. (2016). Construction of high resolution genetic linkage maps to improve the soybean genome sequence assembly Glyma1.01. BMC Genom..

[30] Robinson H.F., Comstock R.E., Harvey P.H. (1949). Estimates of Heritability and the Degree of Dominance in Corn 1. Agron. J..

[31] Maranna S., Verma K., Talukdar A., Lal S.K., Kumar A., Mukherjee K. (2016). Introgression of null allele of Kunitz trypsin inhibitor through marker-assisted backcross breeding in soybean (*Glycine max* L. Merr.). BMC Genet..

[32] Vineet K., Rani A., Rawal R., Mourya V. (2015). Marker Assisted Accelerated In-trogression of Null Allele of Kunitz Trypsin Inhibitor in Soybean. Breed. Sci..

[33] Taishi U., Sakurai T., Totoki Y., Toyoda A., Seki M., Ishiwata A., Akiyama K., Kurotani A., Yoshida T., Mochida K. (2008). Sequencing and Analysis of Approximately 40,000 Soybean Cdna Clones from a Full-Length-Enriched Cdna Library. DNA Res. Int. J. Rapid Publ. Rep. Genes Genomes.

[34] Rashed N.A., Macdonald M.H., Matthews B.F. (2008). Protease Inhibitor Expres-sion in Soybean Roots Exhibiting Susceptible and Resistant Interactions with Soybean Cyst Nema-tode. J. Nematol..

[35] Riascos J. (2009). A Genomics-Based Search for Novel Soybean Allergens. Ph.D. Thsis.

[36] Yuan J., Wen Z., Gu C., Wang D. (2017). Introduction of High Throughput and Cost Effective SNP Genotyping Platforms in Soybean. Plant Genet. Genom. Biotechnol..

[37] Patil G., Chaudhary J., Vuong T.D., Jenkins B., Qiu D., Kadam S., Shannon G.J., Nguyen H.T. (2017). Development of SNP Genotyping Assays for Seed Composition Traits in Soybean. Int. J. Plant Genom..

[38] Kadam S., Vuong T.D., Qiu D., Meinhardt C.G., Song L., Deshmukh R.K., Patil G., Wan J., Valliyodan B., Scaboo A.M. (2016). Genomic-assisted phylogenetic analysis and marker development for next generation soybean cyst nematode resistance breeding. Plant Sci..

[39] Rosso M.L., Shang C., Correa E., Zhang B. (2018). An Efficient HPLC Approach to Quantify Kunitz Trypsin Inhibitor in Soybean Seeds. Crop. Sci..

[40] Saghai-Maroof M.A., Soliman K.M., Jorgensen R.A., Allard R.W. (1984). Ribosomal DNA spacer-length polymorphisms in barley: Mendelian inheritance, chromosomal location, and population dynamics. Proc. Natl. Acad. Sci. USA.

[41] Song Q., Hyten D.L., Jia G., Quigley C.V., Fickus E.W., Nelson R.L., Cregan P.B. (2013). Development and Evaluation of SoySNP50K, a High-Density Genotyping Array for Soybean. PLoS ONE.

[42] Van Ooijen J.W. (2006). Joinmap 4 ®: Software for the Calculation of Genetic Linkage Maps. Experimental Populations.

[43] Kosambi D.D. (1943). The Estimation of Map Distances from Recombination Values. Ann. Eugen..

[44] Meng L., Li H., Zhang L., Wang J. (2015). Qtl Icimapping: Integrated Software for Genetic Linkage Map Construction and Quantitative Trait Locus Mapping in Biparental Populations. Crop J..

[45] Voorrips R.E. (2002). MapChart: Software for the Graphical Presentation of Linkage Maps and QTLs. J. Hered..

[46] Zhu Q., Escamilla D.M., Wu X., Song Q., Li S., Rosso M.L., Lord N., Xie F., Zhang B. (2020). Identification and validation of major QTLs associated with low seed coat deficiency of natto soybean seeds (*Glycine max* L.). Theor. Appl. Genet..

[47] Qijian S., Hyten D.L., Jia G., Quigley C.V., Fickus E.W., Nelson R.L., Cregan P.B. (2015). Fingerprinting Soybean Germplasm and Its Utility in Genomic Research. G3: Genes Genomes Genet..

[48] Nyquist Wyman E., Baker R.J. (1991). Estimation of Heritability and Prediction of Selection Re-sponse in Plant Populations. Crit. Rev. Plant Sci..

